# Dynamic interplay between H-current and M-current controls motoneuron hyperexcitability in amyotrophic lateral sclerosis

**DOI:** 10.1038/s41419-019-1538-9

**Published:** 2019-04-05

**Authors:** Yossi Buskila, Orsolya Kékesi, Alba Bellot-Saez, Winston Seah, Tracey Berg, Michael Trpceski, Justin J. Yerbury, Lezanne Ooi

**Affiliations:** 1 Biomedical Engineering and Neuroscience research group, The MARCS Institute, Western Sydney University, Penrith, NSW 2751 Australia; 2 School of Medicine, Western Sydney University, Campbelltown, NSW 2560 Australia; 3 School of Chemistry and Molecular Bioscience, University of Wollongong, Northfields Avenue, Wollongong, NSW 2522 Australia; 4 Illawarra Health and Medical Research Institute, Northfields Avenue, Wollongong, NSW 2522 Australia

## Abstract

Amyotrophic lateral sclerosis (ALS) is a type of motor neuron disease (MND) in which humans lose motor functions due to progressive loss of motoneurons in the cortex, brainstem, and spinal cord. In patients and in animal models of MND it has been observed that there is a change in the properties of motoneurons, termed neuronal hyperexcitability, which is an exaggerated response of the neurons to a stimulus. Previous studies suggested neuronal excitability is one of the leading causes for neuronal loss, however the factors that instigate excitability in neurons over the course of disease onset and progression are not well understood, as these studies have looked mainly at embryonic or early postnatal stages (pre-symptomatic). As hyperexcitability is not a static phenomenon, the aim of this study was to assess the overall excitability of upper motoneurons during disease progression, specifically focusing on their oscillatory behavior and capabilities to fire repetitively. Our results suggest that increases in the intrinsic excitability of motoneurons are a global phenomenon of aging, however the cellular mechanisms that underlie this hyperexcitability are distinct in SOD1^G93A^ ALS mice compared with wild-type controls. The ionic mechanism driving increased excitability involves alterations of the expression levels of *HCN* and *KCNQ* channel genes leading to a complex dynamic of H-current and M-current activation. Moreover, we show a negative correlation between the disease onset and disease progression, which correlates with a decrease in the expression level of *HCN* and *KCNQ* channels. These findings provide a potential explanation for the increased vulnerability of motoneurons to ALS with aging.

## Introduction

### Motor neuron disease and excitability

Amyotrophic lateral sclerosis (ALS) is the most prevalent form of motor neuron disease (MND) and is characterized by a progressive loss of both upper and lower motor neurons, leading to paralysis and ultimately death due to a respiratory failure, often within 3–5 years from diagnosis. In the absence of a unifying mechanism that leads to the clinical symptoms, multiple processes have been found to be instrumental in disease progression. One of the most prominent changes in MND patients is an increased excitability of motoneurons, leading to fasciculation, cramps, hyperreflexia, and spasticity in MND patients^1^. Due to these symptoms, it has been hypothesized that neuronal hyperexcitability mediated by glutamate excitotoxicity is a leading cause for neuronal loss during MND, reviewed by ref. ^2^. However, recent studies have challenged this concept, questioning the impact of increased cellular excitability on neuronal survival^3^, and influence on excitability symptoms^4,5^. Moreover, recent in vivo studies showed that adult spinal motoneurons are not hyperexcitable^6^, and that fast fatigable lower motoneurons, which are most vulnerable in amyotrophic lateral sclerosis mouse models, display hypoexcitability and loss of repetitive firing rather than hyperexcitability prior to degeneration^7^.

### SOD1 mouse model

Familial ALS (fALS) accounts for approximately 5–10% of all ALS cases. The first discovery linked to fALS was a mutation in the ubiquitously expressed Cu–Zn superoxide dismutase (SOD1) enzyme, which catalyzes the dismutation of superoxide anions (O_2_^−^) to oxygen and H_2_O_2_^8^. Indeed, mutant SOD1 has been found to cause malfunction of many cellular pathways and processes, including excitotoxicity and mitochondrial stress, which leads to cell death^9^. Identification of SOD1 mutations in ALS led to the development of several mouse models, including a transgenic mouse that expresses multiple copies of a mutated form of human SOD1 (SOD1^G93A^), and that develops a progressive ALS with many similarities to the human disease^10^.

### Aims of study

Neuronal excitability is not a static phenomenon but rather shows a pattern of progression in a spatiotemporal aspect^1^, in which the biophysiological properties of motoneurons change with age. Therefore, it is essential to distinguish between physiological alterations in cellular excitability that are simply due to normal aging and alterations that are caused by the progression of the disease. Previous studies examining hyperexcitability in ALS mouse models have used mainly embryonic or early postnatal stage neurons, which has made it difficult to assess the role of hyperexcitability in normal aging. Hence, the aim of this study was to monitor the physiological properties of upper motoneurons during aging, as well as in different stages of the disease, which is crucial for our understanding of the mechanisms leading to motoneuron degeneration.

## Results

To assess cellular alterations occurring during the progression of ALS, we recorded the electrophysiological properties of upper motoneurons (UMNs) residing in layer 5b of the motor cortex of transgenic mice expressing the mutated human *SOD1* gene (SOD1^G93A^) and their wildtype littermate controls^10^. Mice were divided into two different age groups, corresponding to definite disease stages. The first group consisted of SOD1 mice at the age of 70–80 days (average of 73 ± 1 days). Although previous reports indicate that these mice show cellular differences as early as a few days^3,11^, our mouse colony at this age were still lacking behavioral phenotypes or any motor dysfunction, so we named them the ‘Young SOD1′ group (*n* = 19). The second group, ‘Aged SOD1′ were SOD1 mice at the age of 5–7 months, which showed symptoms of ALS (Symptomatic group, *n* = 55), including rigid paralysis and minimal joint movement of the limbs, corresponding to neurological score (NS) 2 as described by refs. ^12,13^. As controls for normal aging, we used age-matched littermates named ‘Young Control’ (*n* = 21) and ‘Aged Control’ (*n* = 53), which neither express the mutant human SOD1 enzyme, nor show any symptoms.

### Characteristics of our mouse colony

**Fig. 1:**
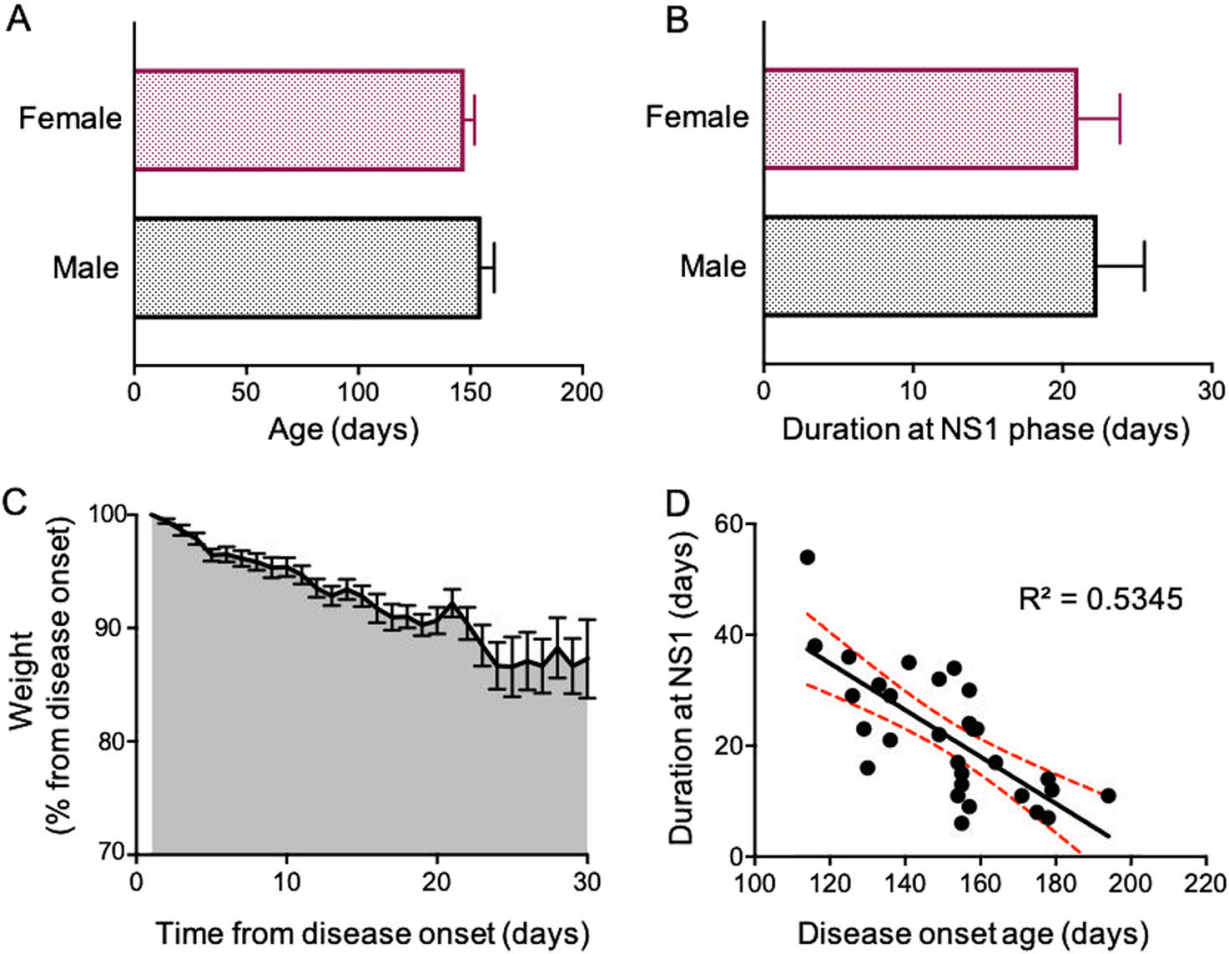
Assessment of motor function in SOD1 mice colony. **a** Bar graph depicting the average age in which SOD1 symptomatic mice showed deterioration in motor function (NS 1 according to criteria outlined by the ALS therapy Development Institute). **b** Bar graph depicting the average duration of SOD1 males and females at the NS 1 phase. **c** A plot depicting the change in body weight (for both males and females) as a percentage of the body weight measured on the first day of disease onset. Data in **a**–**c** is presented as mean ± S.E.M. **d** Plot of the duration at NS 1 phase vs the age of disease onset depicting moderate negative correlation. The fitted regression line is shown with 95% mean prediction interval

Our mouse colony consisted of 55 symptomatic mice, 28 males and 27 females. While the average age in which females started showing deterioration in motor function (NS 1; corresponding to motor weakness—depicted as partial collapse of leg extension or trembling of hind legs during tail suspension) was 147 ± 4 days, the males started showing symptoms at the age of 155 ± 5 days (Fig. 1a). On average, SOD1 mice reached NS 2 after 168 ± 2 days, (no significant differences between genders, student *t*-test), which is longer than the period of 17–20 weeks published in previous reports^10,14^. Moreover, the deterioration rate from the first onset of the symptoms until the mice reached NS 2 was longer than previously described, averaging 21 ± 2 days in females and 22 ± 3 days in males (Fig. 1b). These alterations in motor function were accompanied with weight loss (Fig. 1c), as previously described by other studies^12,14^.

Intriguingly, there was a moderate negative correlation between the disease onset (the age at which the mice first showed symptoms of motor impairment (NS 1)) and the duration in which the mice stayed at the NS 1 phase before progressing to NS 2 (Fig. 1d). These changes in disease progression from previous studies are most likely attributed to a decrease in the relative copy number of the expressed human *SOD1* transgene, (6.2 ± 0.2, see^12^) compared with the original SOD1G93A mouse line^10,15^ that reportedly had a gene copy number of 18 ± 2.6.

### Cortical motoneurons from symptomatic SOD1 mice are hyperexcitable

To assess alterations in the intrinsic properties of cortical motorneurons during disease progression, we have recorded the passive and active electrophysiological properties of pyramidal neurons residing in layer 5b of the primary motor cortex (M1). Layer 5 pyramidal neurons are heterogenous and consist of various types of motoneurons, including corticospinal and corticostriatal motoneurons, which display different electrophysiological properties^16–19^. Two of the most prominent features of corticospinal neurons are their distinct laminar location in layer 5b of the motor cortex and relatively high Sag amplitude, ranging from 10 to 40%, as previously reported by refs. ^16–19^. Although previous reports showed that the overall excitability profile of all cortical neurons increased in SOD1 mice^4,20^, to reduce bias selection of the recorded neurons, only pyramidal neurons residing in layer 5b and expressing a Sag amplitude higher than 10% were included in the analysis (Supplementary Fig. 1). The impact of aging and SOD1 mutation on cortical motoneuron excitability was assessed using two-way analysis of variance (ANOVA), followed by a Tukey’s post hoc test. Table 1 summarizes the significant impact of aging, the SOD1 mutation and the interaction between these factors, on the intrinsic membrane properties of cortical motoneurons.

**Table 1:** Age and SOD1 mutation affects the intrinsic properties of cortical motoneurons

	Rin	Rheobase	Sag amplitude	mAHP	*HCN1*	*HCN2*	*HCN3*	*KCNQ2*	*KCNQ3*	*KCNQ5*
Aging	*	****			****	**		****	**	*
SOD1 Mutation			***		***	*	*	**	**	**
Interaction		*		*						

Intrinsic properties of cortical motoneurons change during normal aging and are affected by the SOD1 mutation. Significance levels presented as *p < 0.05, **p < 0.005, ***p < 0.001, ****p < 0.0001; Reported *P*-values are by two-way ANOVA with factors ‘age’ (black asterisks), ‘SOD1 mutation’ (blue asterisks) and interaction between factors (red asterisks)

**Fig. 2:**
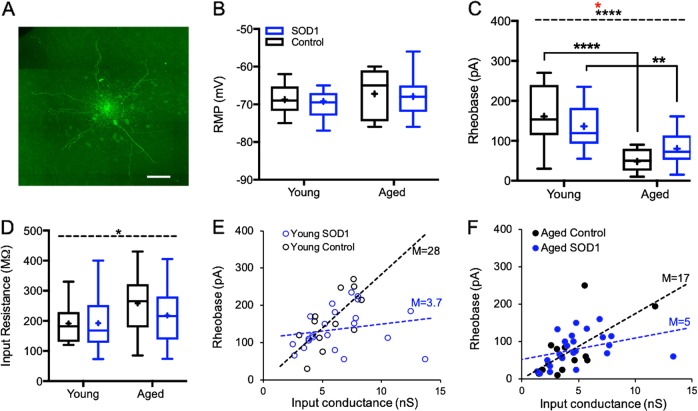
Intrinsic membrane properties of upper motoneurons displaying increased excitability with age. **a** Image of recorded motoneuron filled with biocytin during intracellular recording. Scale bar = 40 μm. **b**–**d** Box plots depicting the distribution of the resting membrane potential (RMP; **b**), rheobase (**c**) and input resistance (**d**) of cortical motoneurons recorded from both SOD1 mice (Young and Aged, coded in blue) and age matched controls (black). The box upper and lower limits are the 25th and 75th quartiles, respectively. The whiskers depicting the lowest and highest data points, while the + sign represents the mean and the horizontal line through the box is the median. Both ‘aged SOD1′ and age matched control mice express a significant decrease in spike rheobase, indicating a global phenomenon occurring during normal aging (black asterisks above the dashed line). **e**, **f** Scatter plots depicting the relationship between the rheobase current and the overall input conductance in the different groups. Each plot was fitted with a linear regression line. The gain of each slope (M) defines the overall excitability profile for each group, indicating an enhanced excitability of SOD1 mice compared to their age matched controls. Asterisks above the dashed line in **c** and **d** represent the significance levels of the impact of “Age” (black), ‘SOD1 Mutation’ (blue) and level of interaction (red). Asterisks below the dashed line represent significance levels between groups following Tukey’s post hoc test. *p < 0.05; **p < 0.005; ***p < 0.001; ****p < 0.0001, two-way ANOVA

On average, the resting membrane potential of layer 5b pyramidal neurons recorded from the primary motor cortex (M1) was comparable between all groups tested (*p* > 0.05; two-way ANOVA, Fig. 2b). Conversely, the excitability of pyramidal neurons from aged SOD1 symptomatic mice was higher than young SOD1 pre-symptomatic mice, depicted as a significant decrease (*F*_(1,65)_ = 40.93, *P* < 0.0001 for the factor aging and *F*_(1, 65)_ = 4.586, *P* < 0.01 for the factor interactions, two-way ANOVA) in the spike rheobase (from 136 ± 15 pA to 80 ± 10 pA; *n* = 22 and 24, respectively, *p* < 0.01, two-way ANOVA with Tukey’s post hoc test; Fig. 2c) and a trend for increased input resistance (from 193 ± 21 MΩ in ‘Young SOD1′ to 218 ± 30 MΩ in ‘Aged SOD1′; Fig. 2d). The increase in cellular excitability was also significant in age-matched controls, as the rheobase significantly decreased from 161 ± 18 pA (*n* = 12) in the ‘Young control’ group to 48 ± 17 pA in the ‘Aged control’ group (*n* = 11, *F*_(1, 65)_ = 4.586, *p* < 0.001, two-way ANOVA with Tukey’s post hoc test). However, closer investigation into the relationship between the rheobase current and the overall input conductance suggested that the excitability profile of cortical motoneurons was higher in the SOD1 mice than their aged-matched controls (depicted as a lower gain of slope, Fig. 2e, f), indicating an impactful involvement of the SOD1 mutation, which interacts with normal aging (Table 1).

**Fig. 3:**
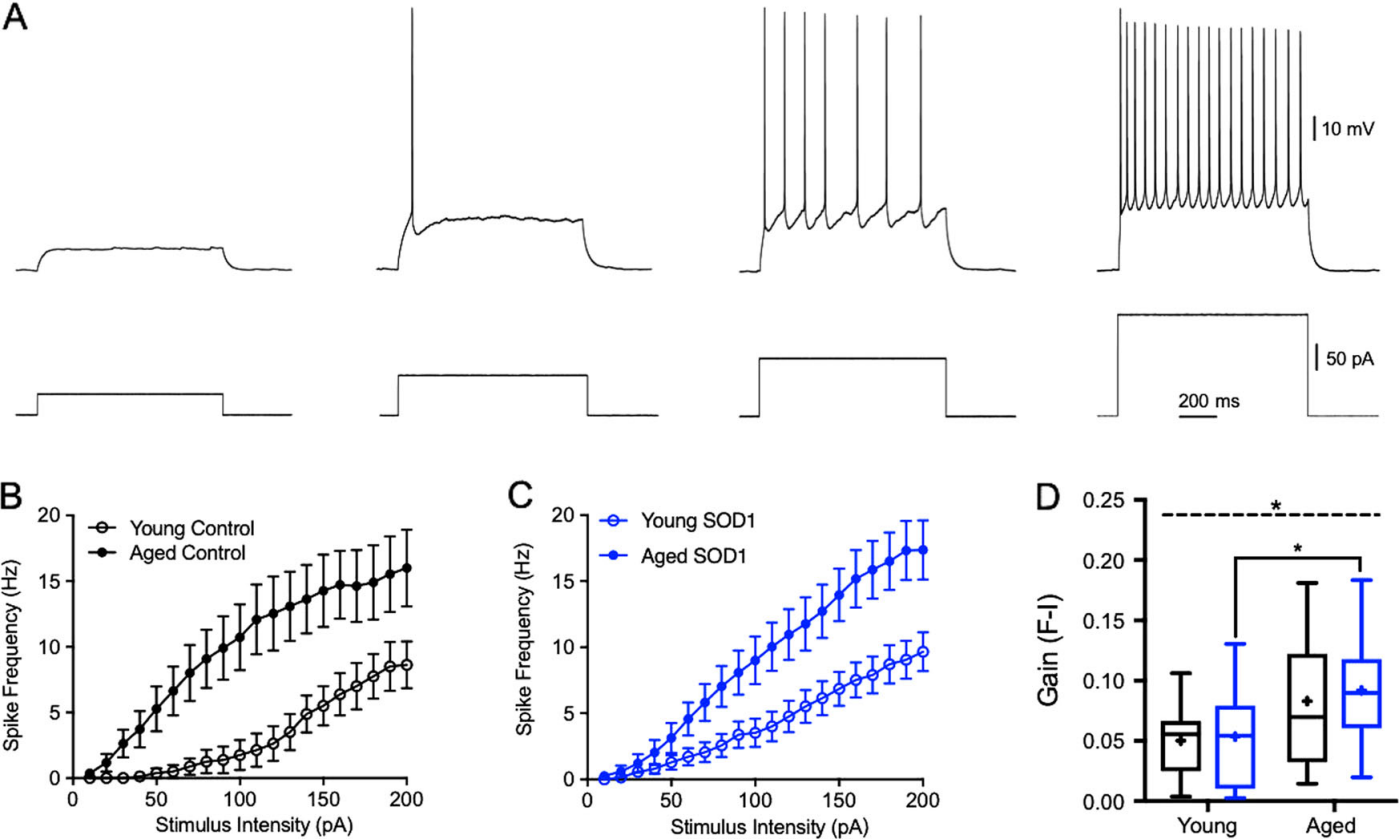
Cortical motoneurons are hyperexcitable in SOD1 symptomatic mice. **a** Representative voltage traces of action potentials evoked by injections of increasing step currents into the soma of cortical motoneurons. **b**, **c** Plots of the spike frequency-current relationship (F-I curves) measured from littermate controls (**b**) and SOD1 mice (**c**) depicting alterations of the spiking trajectories, indicating hyperexcitability of motoneurons recorded from the ‘aged control’ and ‘aged SOD1′ symptomatic group, compared to motoneurons recorded from the ‘Young control’ and ‘Young SOD1′ pre-symptomatic mice respectively. **d** Bar graph depicting the average gain of F-I curves in the different groups. Note the significant impact of aging (asterisk above dash line, two-way ANOVA) on the overall increase in F-I gain, indicative of increased excitability. *p < 0.05; Asterisk below dashed line represent significance levels between groups using Tukey’s post hoc test. Box plots definition are the same as in Fig. 2

**Fig. 4:**
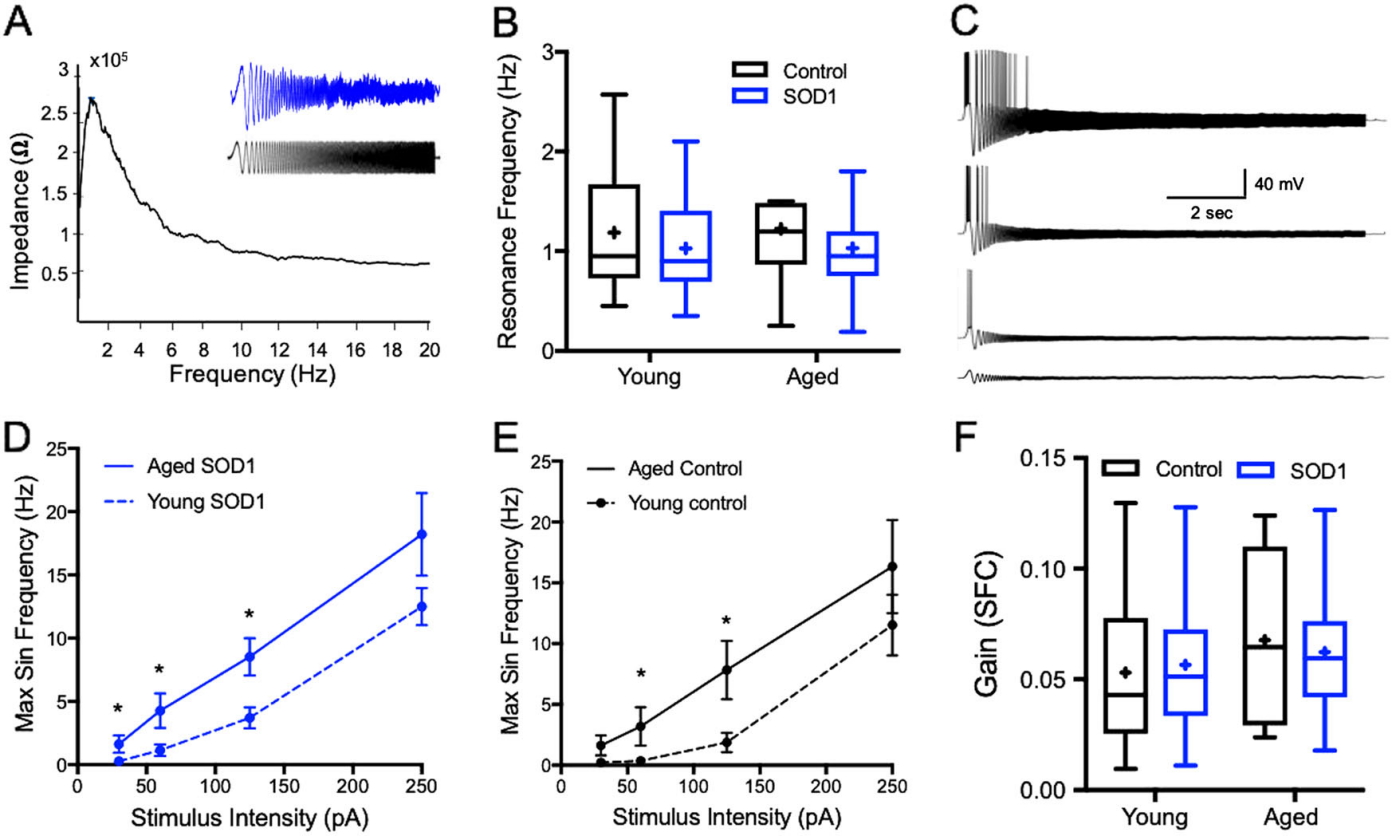
Oscillatory behavior of cortical motoneurons. **a** Impedance amplitude profile depicting the peak resonance frequency of neuron membrane potential (inset, blue trace) following chirp stimulation (inset, black trace). **b** Box plots depicting the distribution of the resonance frequency in all groups tested. **c** Sample voltage traces recorded following sinusoidal chirp stimulation (0.1–100 Hz) at different intensities (from bottom to top: 30, 60, 125, 250 pA), which were used to build the sinusoidal-frequency spiking curves. **d**, **e** Sinusoidal-frequency curves depicting the relationship between the oscillation intensity and the maximal frequency at which the cell is still excitable. Note the upward shift in the maximal oscillation frequency in both aged control (**d**) and symptomatic mice (**e**), compared to ‘young control’ and ‘young SOD1′ pre-symptomatic mice respectively. *p < 0.05; Mann-Whitney test. **f** Box plots depicting the distribution of the gain of the sinusoidal-frequency curves in all groups tested. Box plots definition are the same as in Fig. 2

Neuronal spiking activity is determined by a complex interaction of voltage dependent inward and outward currents, and underlies the execution of neuronal output which contributes significantly to network behavior^21^. As muscle movement is defined by the firing rate of motoneurons, and one of the hallmarks of ALS is a loss of the ability of lower motoneurons to fire repetitively^6,7^, we measured the firing frequency-current (F-I) curves (Fig. 3a–c), as well as the oscillatory behavior (Fig. 4) of cortical motoneurons. Our results showed that the average gain of the spiking F-I curves of motoneurons from symptomatic “aged SOD1” mice was significantly higher (*F*_(1, 57)_ = 9.442, *P* < 0.001, two-way ANOVA) than pre-symptomatic “young SOD1” motoneurons (0.09 ± 0.01 Hz/pA, *n* = 21 vs. 0.05 ± 0.01 Hz/pA, *n* = 21 respectively; *p* < 0.05; two-way ANOVA with Tukey’s post hoc test; Fig. 3c, d), indicating an increased excitability and ability to fire at higher frequencies. However, there was a trend for an increase in the average gain of F-I curves recorded from aged controls compared with young controls (Fig. 3b), though this did not reach statistical significance.

Neurons act as resonators that on the one hand respond preferentially to inputs at a certain frequency, and on the other affect the network oscillatory activity^22^. Neuronal resonance frequency is determined by the interplay of passive and active properties^23,24^, mainly slowly activating K^+^ current and a fast-persistent Na^+^ current^25^, and affects spike propagation and adaptation^23^. Our results show that the average resonance frequency of the cortical motoneurons recorded remained stable and neither change due to normal aging nor during disease progression (Fig. 4a, b).

In order to evaluate the relationship between membrane oscillation frequencies and spike threshold, we injected sinusoidal currents at different intensities (30–250 pA chirp current) at increasing frequencies (0.1–100 Hz; Fig. 4c). This protocol detects neuronal excitability at instantaneous sinusoidal frequencies and allows an evaluation of the relationship between neuronal excitability and oscillatory behavior, depicted by the Frequency-Spiking Curve (SFC, Fig. 4d, e). Analysis of the sinusoidal frequency curves of upper motoneurons suggest that following low amplitude sinusoidal stimulation (≤125 pA), motoneurons from both ‘aged control’ mice and ‘aged SOD1′ symptomatic mice were able to fire action potentials at higher oscillations frequencies than young mice (7.8 ± 2.3 Hz (*n* = 8) and 8.5 ± 1.4 Hz (*n* = 23) vs. 1.8 ± 0.8 Hz (*n* = 12) and 3.7 ± 0.8 Hz (*n* = 22), respectively; *U* = 17, *p* < 0.01 for control and *U* = 128, *p* < 0.001 for SOD1, Mann-Whitney’s two-tailed *U*-test, Fig. 4d, e). However, no significant alterations were identified at higher amplitudes (>125 pA), indicating a differential impact of aging on the maximal firing frequency. Moreover, no differences were observed between the gain of SFCs recorded from SOD1 mice and their age-matched controls (Fig. 4f). Together these results suggest that the intrinsic alterations controlling the maximal frequency at which neurons were still able to fire action potentials, were limited to lower spiking frequencies and were simply due to the physiological impact of aging.

### Cortical neurons lose their capability of repetitive firing in pre-symptomatic stages

**Fig. 5:**
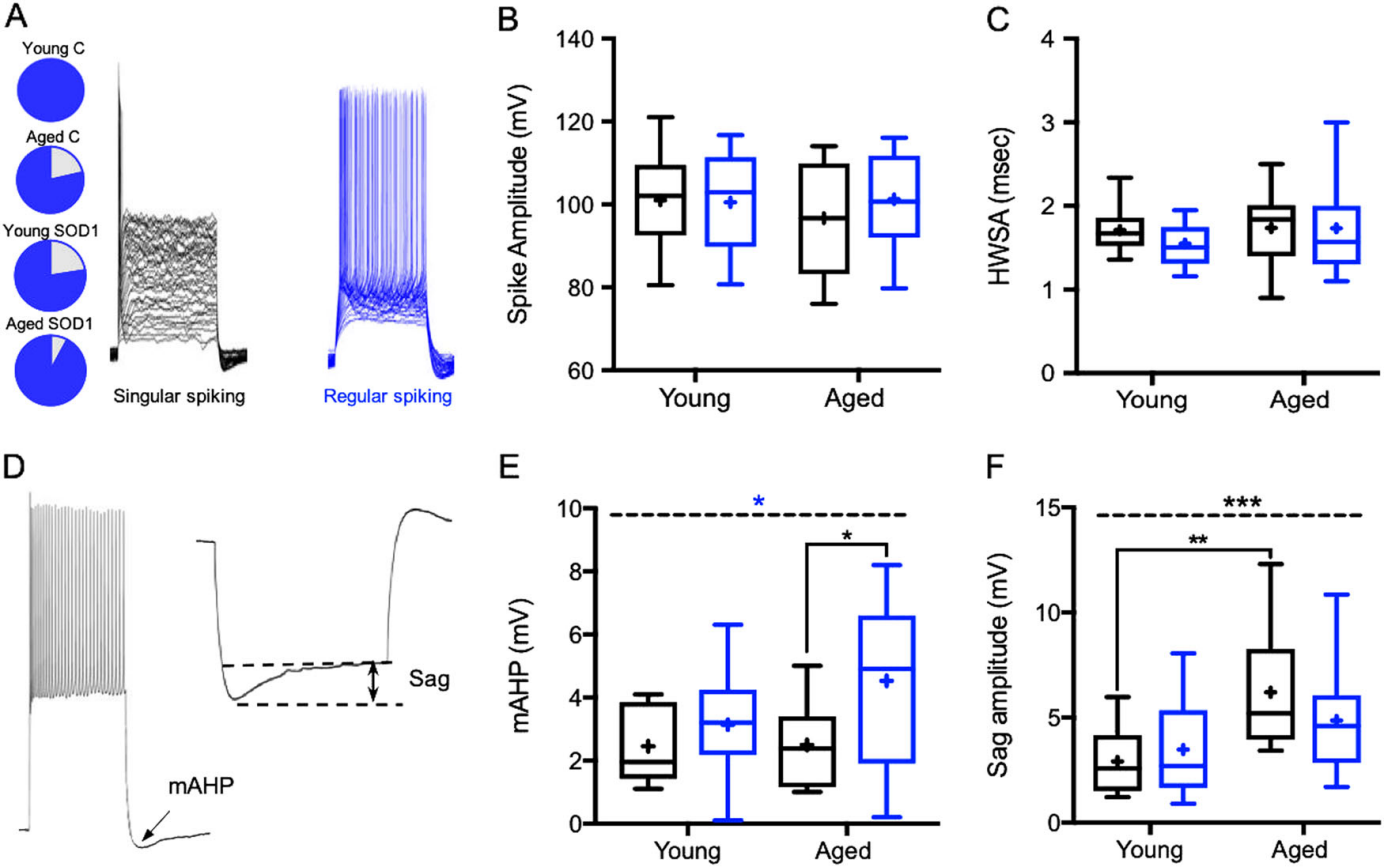
Motor neurons lose their capability of repetitive firing during pre-symptomatic stages. **a** Pie chart depicting the portion of neurons that cannot fire repetitively out of all recorded pyramidal neurons. Sample traces of singular (left) and regular (right) spiking neurons. **b**, **c** Box plots depicting the distribution of the spike amplitude (**b**) and spike width at half amplitude (SWHA; C) in the different groups. Box plots definition are the same as in Fig. 2, no significant differences were noted between groups. **d** Sample traces depicting the mAHP amplitude (left) recorded following train of action potentials, and Sag amplitude (right) recorded following administration of hyperpolarizing current. **e** Box plots describing the distribution of the mAHP, indicating a significant increase in mAHP amplitude of ‘Aged SOD1′ symptomatic mice compared to ‘Young SOD1′ pre-symptomatic animals, which is due to SOD1 mutation. **f** Box plots of the Sag amplitude, as indication for *I*_*h*_ conductance, identify significant increase which is due to aging. Asterisks above the dashed line in **e** and **f** represent the significance levels of the impact of “Age” (black) and ‘SOD1 Mutation’ (blue). Asterisks below the dashed line represent significance levels between groups following Tukey’s post hoc test. *p < 0.05; **p < 0.005; ***p < 0.001; two-way ANOVA

Previous reports suggested that in ALS, neurons lose their capability of repetitive firing, and once symptoms of ALS appear, a substantial amount of motoneurons may have already degenerated^26^. This implies that most neuronal death occurs during the pre-symptomatic stages and progress with time. For this reason, we compared the spiking activity of all pyramidal neurons recorded in layer 5 of the motor cortex. In order to assess neuronal firing patterns, we injected increasing step currents greater than >150% of rheobase amplitude. All recorded upper motoneurons from young controls showed repetitive firing (Fig. 5a), however 23.0% of upper motoneurons recorded from the ‘young SOD1′ pre-symptomatic mice, lost their capability of repetitive firing. In comparison, only 7.9% of neurons from the ‘aged SOD1′ symptomatic group lost their capability of repetitive firing, while 21.4% of aged controls showed similar features (Fig. 5a). This suggests that at least two-thirds of the neurons that had lost their ability to fire repetitively in the pre-symptomatic stage had been lost by the symptomatic stage of the disease. Further analysis of the spiking properties showed that while the spike amplitude and spike width at half amplitude (SWHA) were similar among all groups (Fig. 5b, c), the medium after hyperpolarization (mAHP; Fig. 5d), which is mediated by small conductance potassium channels^27^, as well as muscarinic and HCN channels^28,29^, increased significantly from 2.5 ± 0.4 mV (*n* = 10) in ‘aged control’ mice to 4.5 ± 0.5 mV (*n* = 24) in ‘aged SOD1′ symptomatic mice (*F*_(1,60)_ = 6.15, *p* < 0.05; two-way ANOVA followed by Tukey’s post hoc test, Fig. 5e). This increase was absent in young mice, suggesting it is specifically attributed to the disease progression in SOD1 symptomatic mice (*F*_(1,60)_ = 6.15, *P* < 0.05, two way ANOVA).

**Fig. 6:**
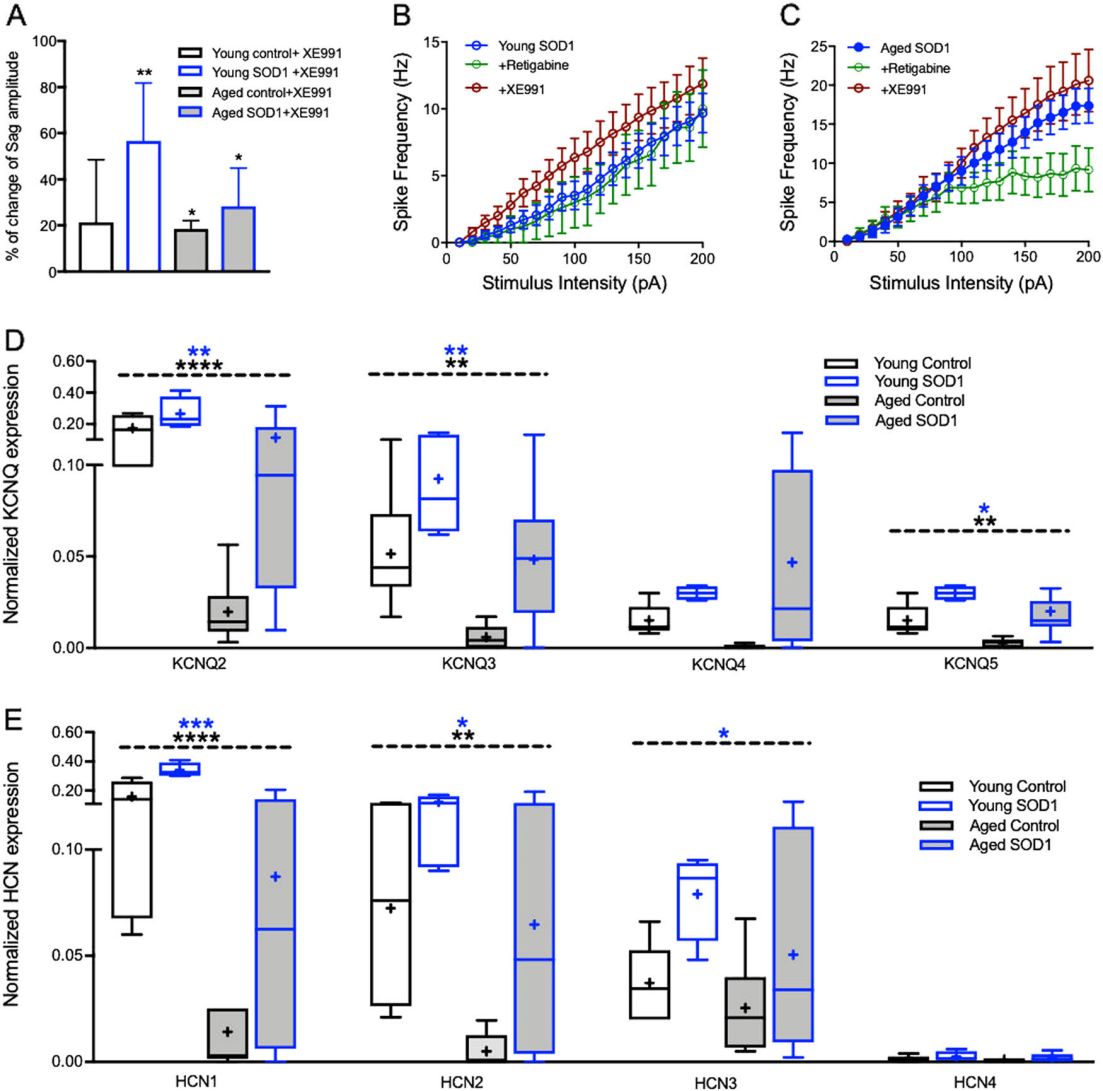
Differential impact of M-current on neuronal excitability. **a** Bar graph depicting the impact of M-current inhibition (via bath application of XE991) on the average Sag amplitude. Although the Sag amplitude increased in all groups, the relative average increase in the ‘young SOD1′ pre-symptomatic group was highest. Data presented as the average % of change of the Sag amplitude recorded prior to application of XE991; *p < 0.05, paired two tailed student *t*-test. **b**, **c** F–I curves following inhibition (+XE991) or facilitation (+Retigabine) of the M-current in ‘young SOD1′pre-symptomatic (**c**) and ‘aged SOD1′ symptomatic (**d**) mice. Note that facilitation of M-current with retigabine in the pre-symptomatic mice had no impact on neuronal excitability. **d** Box plots depicting the distribution of mRNA levels of KCNQ channel genes in the motor cortex normalized to the housekeeper genes. Expression levels of *KCNQ* channel genes are significantly higher in the motor cortex of pre-symptomatic ‘Young SOD1′ mice than symptomatic ‘Aged SOD1′ mice, supporting the relative impact of M-current on the Sag amplitude of pre-symptomatic mice. **e** Box plots depicting the distribution of mRNA levels of *HCN* channel genes in the motor cortex normalized to the housekeeper genes. Box plots definition are the same as in Fig. 2. Asterisks above the dashed line in **d** and **e** represent the significance levels of the impact of “Age” (black) and ‘SOD1 Mutation’ (blue). *p < 0.05; **p < 0.005; ***p < 0.001; ****p < 0.0001, two-way ANOVA

To assess the ionic mechanisms underlying the alterations in neuronal firing patterns and mAHP we measured the impact of H-current (*I*_*h*_) and M-current (*I*_*m*_) on neuronal excitability during disease progression. The H-current is activated by hyperpolarization of the resting membrane potential and mediated by hyperpolarization-activated cyclic nucleotide-gated (HCN) channels, which are permeable for both Na^+^ and K^+^ ions and therefore have an equilibrium potential of −30 mV. This current affects the neuron oscillatory activity, responsiveness to inhibitory synaptic potentials (IPSPs) and resting membrane potential^30^. Analysis of the hyperpolarization-‘Sag’ amplitude (Fig. 5d), mainly mediated by the H-current (*I*_*h*_), identified a significant increase in the Sag amplitude (*F*_(1,64)_ = 15.33, *P* < 0.001, two way ANOVA), which was mainly caused by aging (Fig. 5f, Table 1). However, an increase in *I*_*h*_ conductance is usually accompanied by depolarization of the resting membrane potential and a decrease in input resistance, which we did not observe (see Fig. 2), suggesting other processes may be superimposing and masking our results. For these reasons, we repeated the recordings with a selective antagonist of the non-inactivating K^+^ channels mediating the M-current (*I*_*m*_), XE991^31^. These recordings indicated a significant increase in the Sag amplitude and overall excitability of motoneurons following *I*_*m*_ inactivation, across all groups (*p* < 0.01, *t* = 3.22, df = 14 for young SOD1; *p* < 0.01, *t* = 27, df = 1 for aged control and *p* < 0.05, *t* = 2.57, df = 10 for aged SOD1, two tailed paired student *t*-test, Fig. 6a). Moreover, examination of the F-I curves following inhibition of the M-current led to an upward shift of the F-I curve of motoneurons recorded from both ‘young SOD1′ and ‘aged SOD1′ mice, suggesting enhanced excitability (Fig. 6b, c). In contrast, application of the Kv7 K^+^ channel opener, retigabine, did not affect the F-I curve of motoneurons recorded from ‘young SOD1′ pre-symptomatic mice (Fig. 6b, green markers), yet reduced the excitability of motoneurons recorded from ‘aged SOD1′ symptomatic mice (Fig. 6c). The relative change in Sag amplitude following *I*_*m*_ inhibition was highest in ‘young SOD1′ pre-symptomatic mice (average increase of 57 ± 25% compared with 28 ± 17% in ‘aged SOD1′ symptomatic mice), consistent with the significantly higher expression levels of *KCNQ2* (*F*_(1, 28)_ = 21.08, *P* < 0.0001 for aging and *F*_(1, 28)_ = 7.64, *P* < 0.01 for mutation) and *KCNQ3* (*F*_(1, 28)_ = 11.92, *P* < 0.001 for aging and *F*_(1, 28)_ = 10.29, *P* < 0.001 for mutation) channel genes in the motor cortex of ‘young SOD1′ mice (compared with symptomatic ‘aged SOD1′ animals; *p* < 0.05, two way ANOVA with Tukey’s post hoc test, Fig. 6d). Further analysis of the expression level of *HCN* channel genes in the motor cortex of all groups tested identified a significant decrease of *HCN1* channel expression during both normal aging (*F*_(1, 26)_ = 33.9, *p* < 0.0001, two-way ANOVA) and disease progression (*F*_(1, 26)_ = 13.84, *p* < 0.001, two-way ANOVA, Fig. 6e, Table 1), yet the expression level in SOD1 mice was significantly higher than their littermate controls. Intriguingly, the expression pattern of *HCN1, KCNQ2*, and *KCNQ3* channels was similar, depicting a significant decrease with age, but greater expression in SOD1 mice (Fig. 6d, e), underpinning the differential impact of *I*_*m*_ on cortical motoneurons during symptomatic and pre-symptomatic stages. Together our data thus identifies a complex dynamic between H-current and M-current in aging and in ALS.

## Discussion

ALS is a multi-factorial disease, however increasing evidence suggests that hyperexcitability, which is thought to develop in the motor cortex, is the underlying mechanism leading to loss of motoneurons^1,32,33^. The source of cortical hyperexcitability can be either via alterations in intrinsic properties of upper motoneurons, extrinsic circuitry processes that affect the inhibition-excitation balance^34^, or via modulation of glial activity^9,35^. However, a recent study that investigated the correlation between selective loss of corticospinal neurons and their excitability showed that alterations in cortical excitability are not cell-type specific, but rather a global phenomenon of the motor cortex. Indeed both excitatory and inhibitory neurons exhibited increases in intrinsic excitability, which were provisional to the disease stage and counterbalanced by the cortical circuit^4^.

Here, we assessed the intrinsic cellular properties of cortical motoneurons during disease progression in in vitro slice preparations, which provides mechanical stability and fine control of the extracellular environment. Our results suggest that alteration of the intrinsic excitability of cortical motoneurons is a characteristic phenomenon of aging in this mouse model, as indicated by the decrease in spike rheobase in both SOD1 symptomatic mice and their age-matched controls (Fig. 2c). However, two-way analysis of variance showed that the SOD1 mutation affects cellular processes that increase the excitability profile of cortical motoneurons and interact with normal aging, as summarized in Table 1. Moreover, the overall increase in excitability of cortical motoneurons from SOD1 mice suggest that the degree of augmented excitability *is higher in SOD1 mice* compared with their age-matched littermate controls, as indicated by the declined slope of the rheobase-conductance curves (Fig. 2e, f).

Neuronal firing properties form the basis for the neural code, which regulates numerous operations of the central nervous system, including motor control and complex cognitive processes^36^. A key feature of spiking neurons is their capability to act as resonators that amplify inputs at certain preferential frequencies, shaping their firing patterns^23^. The H-current, mediated by hyperpolarization-activated cyclic nucleotide-gated cation channels, affects neuron resonance behavior, as well as synaptic and dendritic integration, thus regulating spiking output and overall motor control^37^. Indeed, computational models show that enhancement of *I*_*h*_ may control the rate of rhythmic oscillations in neural networks^38^, and recent reports showed that upper motoneurons express high levels of *I*_*h*_^19^. In that regard, our results indicate that the expression level of HCN channels was higher in mice expressing the SOD1 mutation (compared with age-matched controls, Fig. 6e), which surprisingly was not accompanied by an increase in the resonance frequency (Fig. 4b), nor a decrease in the overall input resistance as might be expected. This counterintuitive result suggests that the increase in *I*_*h*_ is superimposed with other mechanisms that mask its function. One candidate that is active during subthreshold membrane potential is the M-current, which forms a non-inactivating outward K^+^ current.

*H-current* and *M-current* have biophysiological properties that counterbalance each other, reviewed by ref. ^39^. While activation of *I*_*h*_ leads to membrane depolarization and increased excitability, activation of *M-current*, mediated by four subunits of the K_V_7 (*KCNQ*) K^+^ channel family^40^, will do the opposite. In general, we found that the expression level of *HCN1, HCN2*, and *HCN3* mRNA, as well as *KCNQ2*, *KCNQ3*, and *KCNQ5* channels in the motor cortex decreased with age and disease progression, yet were higher in SOD1 mice than their age-matched controls (Fig. 6b, e). Moreover, the expression level of these channels was gender independent, as no differences were recorded between males and females.

Our results suggest there is a differential impact of K_V_7 channel activity during disease progression, as the increase in the Sag amplitude following *I*_*m*_ inactivation with XE991 was much greater in the ‘young SOD1′ pre-symptomatic mice than in the symptomatic mice (paired *t*-test, Fig. 6a). Hence, these results suggest that the enhancement of *I*_*h*_ during aging could be caused essentially by reduced activity of K_V_7 channels (Fig. 6). As both M-current and H-current are mediated by voltage gated channels, yet they have different activation curves, this means that their combined impact on intrinsic excitability of motoneurons is complex and varies between different types of neurons and age. While M-current consistently drives K^+^ outflow and thus hyperpolarization, once active, it counterbalances the inward Na^+^ flow of the *I*_*h*_ current^39^, leading to the low Sag amplitude seen in young animals (Fig. 5f). Moreover, the reduced M-current in symptomatic mice (‘Aged SOD1′ compared with pre-symptomatic ‘Young SOD1′ mice) could explain the slight trend for increased overall input resistance (Fig. 2). Consistent with our results, Milan and colleagues found a complex age-related dynamic of the cholinergic system in the spinal cord that appeared completely disrupted in SOD1 motoneurons^41^. Moreover, a recent report showed that the Kv7 channel activator retigabine, which increases K_V_7 channel opening and stabilizes the resting membrane potential, both blocks the hyperexcitability and improves motor neuron survival in vitro, when tested in SOD1 ALS patient motor neurons in cell culture^42^. Consistent with this, our results show that application of retigabine had an inhibitory impact on motoneuron excitability, and a greater effect on motoneurons in the symptomatic ‘aged SOD1′ than the pre-symptomatic ‘young SOD1′ mice (compare 6B and 6C).

Overall, our findings provide insights into the intrinsic physiological properties of cortical motoneurons underlying cortical hyperexcitability, a common phenotype in MND. Despite controversy as to the underlying mechanisms leading to cortical hyperexcitability, it is paramount to emphasize that neuronal intrinsic excitability is not a static phenomenon, and is *constantly changing during normal aging*, as well as different pathologies^1^. However, the nature of these properties will determine the transformation of neural signals into active movement, and knowledge about alterations in these properties is an essential step in unraveling new therapeutic targets. Moreover, the negative correlation between disease onset and disease progression (Fig. 1d) might be due to age-dependent compensatory mechanisms for functional motor control, in which the capacity for compensation decreases with age. This compensatory mechanism might involve molecular mechanisms that slow the neurodegenerative process, reviewed by ref. ^43^, or recruitment of other neurons leading to a greater activation of the network involved in motor control, as suggested by ref. ^44^ for Huntington’s disease. Nevertheless, these observations provide insight into potential therapeutic targets that could alleviate disease symptoms or progression.

## Conclusions

We have identified increased neuronal excitability in ‘normal’ aging in cortical motor neurons and a distinct mechanism for aberrant hyperexcitability beyond ‘normal’ aging in a mouse model of ALS. These changes are mediated by a dynamic interplay between H-current and M-current and provides a potential explanation for increased vulnerability of motor neurons to ALS with aging.

## Material and Methods

### Animals

Transgenic SOD1 mice (SOD1^G93A^)1Gur/J were originally from the colony held by Justin Yerbury (University of Wollongong, NSW, Australia). Mice were bred and maintained on the B6SJL background by breeding mSOD1 transgenic males with non-transgenic females in a rotational scheme. All animals handled with standard conditions of temperature, humidity, 12 h light/dark cycle, free access to food and water, and without any intended stress stimuli. All experiments were approved and performed in accordance with Western Sydney University committee for animal use and care guidelines (Animal Research Authority #A11789).

### Slice preparation

Animals were deeply anesthetized by inhalation of isoflurane (5%). Following anesthesia, mice were transcardially perfused with ice-cold HEPES-buffered N-methyl glucamine-artificial cerebrospinal fluid (aCSF) solution containing in mM: 2.5 KCl; 1.25 NaH_2_PO4; 25 NaHCO_3_; 25 D-glucose; 10 MgSO_4_; 92 NMDG (N-methyl glucamine); 0.5 CaCl_2_; 20 HEPES; 2 Thiourea; 5 Na-ascorbate; 3 Na-pyruvate (NMDG-ACSF), until the outflow solution was clear. After perfusion, the mice were decapitated, and their brains were quickly removed and placed into ice-cold aCSF containing (in mM): 125 NaCl, 2.5 KCl, 1 MgCl_2_, 1.25 NaH_2_PO_4_, 2 CaCl_2_, 25 NaHCO_3_, 25 glucose, and saturated with carbogen (95% O_2_–5% CO_2_ mixture; pH 7.4). Parasagittal brain slices (300 μm thick) were cut with a vibrating microtome (Leica VT1200S) and transferred to the Braincubator^TM^ (PaYo Scientific, Sydney; http://braincubator.com.au), as reported previously^45^. The Braincubator is an incubation system that closely monitors and controls pH, carbogen flow, and temperature, as well as irradiating bacteria through a separate UV chamber^46,47^. Slices were initially incubated for 12 min at 35 °C, after which they were allowed to cool to 15–16 °C and kept in the Braincubator^TM^ for at least 30 min before any measurement^48^.

### Electrophysiological recording and stimulation

The recording chamber was mounted on an Olympus BX-51 microscope equipped with IR/DIC optics. Following incubation in the Braincubator^TM^, slices were mounted in the recording chamber for a minimum of 15 min, to allow them to warm upto room temperature (~22 °C), and were constantly perfused at a rate of 2–3 ml/min with carbogenated aCSF, as reported previously^49^. Whole-cell intracellular recordings from layer V pyramidal neurons in the motor cortex were obtained with patch pipettes (5–7 MΩ) containing (in mM): 130 K-Methansulfate, 10 HEPES, 0.05 EGTA, 7 KCl, 0.5 Na_2_GTP, 2 Na_2_ATP, 2 MgATP, 7 phosphocreatine, and titrated with KOH to pH 7.2 (∼285 mOsm). Voltages were recorded in current clamp mode using a multiclamp 700B dual patch-clamp amplifier (Molecular Devices), digitally sampled at 30–50 kHz, filtered at 10 kHz, and analyzed off-line using pClamp 10 software^23^. Membrane properties were obtained before and after bath application of the KCNQ/K_v7_ channel blocker XE991 (3 µM) or the KCNQ/K_v7_ enhancer retigabine (10 µM), as previously reported^31^. Cells were considered stable and suitable for analysis if the input resistance did not change more than 20% during the baseline recordings, before any treatment.

The Sag potential was measured following injection of hyperpolarizing current steps (−50 to −150, 1 s, Fig. 5d) and determined as the difference between the minimum peak potential and the steady state potential, as previously described^19,37^. Sag potentials were normalized to their respective peak deflection from resting membrane potential, and only neurons with Sag potentials greater than 10% were included in the analysis.

### Suprathreshold sinusoidal stimulus protocol

In order to evaluate alterations in suprathreshold oscillation frequencies under different conditions, 10-s stimulating protocols of sinusoidal current (chirp stimulation), in which there was a linear increase in the frequency from 0.1 to 100 Hz, were designed at 30, 60, 125, and 250 pA, using the pClamp 10 software suite (Molecular devices, Sunnyvale, CA).

### Spectrum impedance analysis

To measure the resonance frequency of individual neurons, a 20-s subthreshold sinusoidal current at 10 pA, with a linear increase in frequency from 0.1 to 20 Hz (chirp stimulation) was applied through the recording electrode, as previously described^23^. The resonance frequency was determined as the peak in the impedance amplitude profile (ZAP) generated by dividing the Fourier transforms of the voltage signal by that of the current signal, as previously described by ref. ^25^. The voltage recordings were recorded in millivolts and the current signal recorded in picoamps and adjusted accordingly such that the resulting complex impedance can be measured in Ohms.

### qRT-PCR

Halved mouse motor cortices for qRT-PCR analysis were stored in 0.5 mL RNA*later* Solution (Ambion) at −80 °C. RNA was extracted using the Qiagen RNeasy Plus Mini Kit, with tissue samples blotted to remove excess RNA*later* Solution before being snap-frozen in liquid nitrogen. Tissues were homogenized using a mini-pestle followed by disruption by passing through a 21 G needle 5 times and extraction as per kit instructions, with RNA eluted in 30 µL volume. RNA yield was determined using a NanoDrop 2000/2000c Spectrophotometer (Thermo Fisher Scientific) and 1 µg of RNA was used per 20 µL cDNA synthesis reaction, using the Tetro cDNA Synthesis Kit (Bioline) with Oligo (dT)_18_ primer. An equivalent dilution of RNA was prepared in water and stored at −20 °C for inclusion in qRT-PCR experiments to assess RNA preparations for genomic contamination (“no RT” control). The SensiFAST™ SYBR No-ROX Kit (Bioline) was employed for qRT-PCR, with 20 µL reactions performed in triplicate. Intron-spanning primers (Table 2; synthesized by Sigma-Aldrich) were selected from PrimerBank^50^ or designed in-house and evaluated via BLAST and Ensembl searches. Reactions contained 0.4 µM final concentration of each primer, along with 50 ng of cDNA. Negative controls included water (NTC) and the “no reverse transcriptase” RNA samples. Three-step cycling was carried out on a QuantStudio 5 Real-Time PCR System (Thermo Fisher Scientific) with conditions of 95 °C for 2 min, then 40 amplification cycles of 95 °C for 5 s, 57–63 °C (primer-dependent; Table 2) for 10 s, and 72 °C for 20 s, followed by a single melt cycle of 95 °C for 15 s, 60 °C for 1 min, then increasing to 95 °C at a rate of 0.1 °C/s. The threshold was automatically set by the QuantStudio Design and Analysis Software (v1.4.3) and the Ct was calculated as the average of the three replicates. The Ct values for transcripts of interest were normalized to the average of the two reference genes, *Ccdc127* and *Hprt* (Table 2).

**Table 2:** List of qPCR primers

Target	Forward primer (5′–3′)	Reverse primer (5′–3′)	Product size (bp)	Annealing temp. (°C)	Primerbank ID or source
Ccdc127	TGGAATTATGCCCTATTGGTGC	TCACAGCATGGTATTTGGCTTC	157	63	281183277c1
Hprt	AGTCCCAGCGTCGTGATTAG	TTTCCAAATCCTCGGCATAATGA	88	61	96975137c1
Hcn1	CAAATTCTCCCTCCGCATGTT	TGAAGAACGTGATTCCAACTGG	182	62	283837798c1
Hcn2	GCTCATCCGATATATCCACC	TGGCAGAGCAGTAGCATC	111	57	n/a
Hcn3	GAGGAGTTCCCAATGATGC	CGTTTCCGCTGCAGTATC	95	59	n/a
Hcn4	ATTGACTCGGAGGTCTACAAAAC	TTCACGATGCGTACCACGG	173	59	124487124c3
Kcnq2	TTTCCACCATCAAGGAGTACGA	CCGAATACCACGATAGTCACGAT	79	62	54873655c2
Kcnq3	CAAGTACAGGCGCATCCAAAC	GGCCAGAATCAAGCATCCCA	115	63	282398105c2
Kcnq4	TTGACGAGTATTCAGCAGGACA	GGACCCTTATCGCCCTTCTC	127	62	7671226a1
Kcnq5	GTCGGCGCAACGTCAAGTA	AACCAAACACAAGGAGAAAACG	114	62	8132999a1

### Statistical analysis

Unless stated, data is reported as mean ± S.E.M. Statistical comparisons were performed with Prism 7 (GraphPad Software; San Diego, CA) using two-way ANOVA followed by Tukey’s post hoc test, or two-tailed paired student *t*-test as detailed in the text. Data in figures are presented in two levels. Asterisks above A dashed line represent the significance levels of the impact of “Age” (black), ‘SOD1 Mutation’ (blue), and level of interaction (red). Asterisks below the dashed line represent significance levels between groups following Tukey’s post hoc test. *p < 0.05; **p < 0.005; ***p < 0.001; ****p < 0.0001. For the box and whisker plots, the box upper and lower limits are the 25th and 75th quartiles, respectively. The whiskers depicting the lowest and highest data points, while the +sign represents the mean and the horizontal line through the box is the median. Resonance frequency analysis was performed using custom code written in Matlab (Mathworks). Probability values <0.05 were considered statistically significant.

## Supplementary information

Supplementary Figure 1

supplementary figure legends

## References

[1] Bae JS, Simon NG, Menon P, Vucic S, Kiernan MC (2013). The puzzling case of hyperexcitability in amyotrophic lateral sclerosis. J. Clin. Neurol..

[2] Ilieva H, Polymenidou M, Cleveland DW (2009). Non-cell autonomous toxicity in neurodegenerative disorders: ALS and beyond. J. Cell. Biol..

[3] Leroy F (2014). Early intrinsic hyperexcitability does not contribute to motoneuron degeneration in amyotrophic lateral sclerosis. eLife.

[4] Kim J (2017). Changes in the excitability of neocortical neurons in a mouse model of amyotrophic lateral sclerosis are not specific to corticospinal neurons and are modulated by advancing disease. J. Neurosci..

[5] Leroy F, Zytnicki D (2015). Is hyperexcitability really guilty in amyotrophic lateral sclerosis?. Neural Regen. Res..

[6] Delestrée N (2014). Adult spinal motoneurones are not hyperexcitable in a mouse model of inherited amyotrophic lateral sclerosis. J. Physiol..

[7] De Lourdes Martınez Silva, M. et al. Hypoexcitability precedes denervation in the large fast-contracting motor units in two unrelated mouse models of ALS. *Elife* 1–17. 10.1002/acr.23532 (2018). 10.7554/eLife.30955 PMC5922970 29580378

[8] Rosen DR (1993). Mutations in Cu/Zn superoxide dismutase gene are associated with familial amyotrophic lateral sclerosis. Nature.

[9] Dion PA, Daoud H, Rouleau GA (2009). Genetics of motor neuron disorders: new insights into pathogenic mechanisms. Nat. Rev. Genet..

[10] Gurney ME (1994). Motor neuron degeneration in mice that express a human Cu,Zn superoxide dismutase mutation. Science..

[11] Fogarty MJ, Noakes PG, Bellingham MC (2015). Motor cortex layer V pyramidal neurons exhibit dendritic regression, spine loss, and increased synaptic excitation in the presymptomatic hSOD1G93A mouse model of amyotrophic lateral sclerosis. J. Neurosci..

[12] Bartlett R, Sluyter V, Watson D, Sluyter R, Yerbury JJ (2017). P2X7 antagonism using Brilliant Blue G reduces body weight loss and prolongs survival in female SOD1 G93A amyotrophic lateral sclerosis mice. PeerJ.

[13] Hatzipetros, T. et al. A quick phenotypic neurological scoring system for evaluating disease progression in the SOD1-G93A mouse model of ALS. *J. Vis. Exp*. 1–6 10.3791/53257 (2015). 10.3791/53257 PMC4692639 26485052

[14] Weydt P, Hong SY, Kliot M, Möller T (2003). Assessing disease onset and progression in the SOD1 mouse model of ALS. Neuroreport.

[15] Dal Canto MC, Gurney ME (1997). A low expressor line of transgenic mice carrying a mutant human Cu,Zn superoxide dismutase (SOD1) gene develops pathological changes that most closely resemble those in human amyotrophic lateral sclerosis. Acta Neuropathol..

[16] Anderson CT, Sheets PL, Kiritani T, Shepherd GMG (2010). Sublayer-specific microcircuits of corticospinal and corticostriatal neurons in motor cortex. Nat. Neurosci..

[17] Yasvoina MV (2013). eGFP expression under UCHL1 promoter genetically labels corticospinal motor neurons and a subpopulation of degeneration-resistant spinal motor neurons in an ALS mouse model. J. Neurosci..

[18] Oswald MJ, Tantirigama MLS, Sonntag I, Hughes SM, Empson RM (2013). Diversity of layer 5 projection neurons in the mouse motor cortex. Front. Cell. Neurosci..

[19] Suter BA, Migliore M, Shepherd GMG (2013). Intrinsic electrophysiology of mouse corticospinal neurons: A class-specific triad of spike-related properties. Cereb. Cortex.

[20] Hande OP (2011). Corticospinal motor neurons and related subcerebral projection neurons undergo early and specific neurodegeneration in hSOD1 G93A transgenic ALS mice. J. Neurosci..

[21] Connors BW, Gutnick MJ (1990). Intrinsic firing patterns of diverse neocortical neurons. Trends Neurosci..

[22] Tohidi V, Nadim F (2009). Membrane resonance in bursting pacemaker neurons of an oscillatory network is correlated with network frequency. J. Neurosci..

[23] Buskila, Y., Morley, J. W., Tapson, J. & van Schaik, A. The adaptation of spike backpropagation delays in cortical neurons. *Front. Cell. Neurosci*. **7**, 192 (2013). 10.3389/fncel.2013.00192 PMC3812867 24198759

[24] Bellot-Saez A (2018). Astrocytic modulation of cortical oscillations. Sci. Rep..

[25] Gutfreund Y, Yarom Y, Segev I (1995). Subthreshold oscillations and resonant frequency in guinea-pig cortical neurons: physiology and modelling. J. Physiol..

[26] Simon NG (2014). Quantifying disease progression in amyotrophic lateral sclerosis. Ann. Neurol..

[27] Sah P (1996). Ca2 + -activated K + currents in neurones: types, physiological roles and modulation. Trends Neurosci..

[28] Gu N, Vervaeke K, Hu H, Storm JF (2005). Kv7/KCNQ/M and HCN/h, but not KCa2/SK channels, contribute to the somatic medium after-hyperpolarization and excitability control in CA1 hippocampal pyramidal cells. J. Physiol..

[29] Storm BYJF (1989). After-hyperpolarization of medium duration. J. Physiol..

[30] Lüthi A, McCormick DA (1998). H-current: properties of a neuronal and network pacemaker. Neuron.

[31] Lombardo, J. & Harrington, M. A. Non-reciprocal mechanisms of up- and down-regulation of spinal motoneuron excitability by modulators of KCNQ/Kv7 channels. *J. Neurophysiol*. jn.00446.2016 10.1152/jn.00446.2016 (2016). 10.1152/jn.00446.2016 PMC5102305 27512022

[32] Vucic S, Kiernan MC (2006). Novel threshold tracking techniques suggest that cortical hyperexcitability is an early feature of motor neuron disease. Brain.

[33] Vucic S, Nicholson GA, Kiernan MC (2008). Cortical hyperexcitability may precede the onset of familial amyotrophic lateral sclerosis. Brain.

[34] Lee JMartin, Chang Q (2012). Inhibitory synaptic regulation of motoneurons: a new target of disease mechanisms in amyotrophic lateral sclerosis. Mol. Neurobiol..

[35] Do-Ha D, Buskila Y, Ooi L (2017). Impairments in motor neurons, interneurons and astrocytes contribute to hyperexcitability in ALS: underlying mechanisms and paths to therapy. Mol. Neurobiol..

[36] Buzsáki G (2010). Neural syntax: cell assemblies, synapsembles and readers. Neuron.

[37] Sheets PL (2011). Corticospinal-specific HCN expression in mouse motor cortex: Ih-dependent synaptic integration as a candidate microcircuit mechanism involved in motor control. J. Neurophysiol..

[38] Olsen OslashH, Nadim F, Calabrese RL (1995). Modeling the leech heartbeat elemental oscillator II. Exploring the parameter space. J. Comput. Neurosci..

[39] Shah M, Huang Z, Martinello K (2013). HCN and K V 7 (M-) channels as targets for epilepsy treatment. Neuropharmacology.

[40] Wang JJ, Li Y (2016). KCNQ potassium channels in sensory system and neural circuits. Acta Pharmacol. Sin..

[41] Milan L (2015). Age-related changes in pre- and postsynaptic partners of the cholinergic C-boutons in wild-type and SOD1G93A lumbar motoneurons. PLoS ONE.

[42] Wainger BJ (2014). Intrinsic membrane hyperexcitability of ALS patient-derived motor neurons. Cell Rep..

[43] Kreiner G (2015). Compensatory mechanisms in genetic models of neurodegeneration: are the mice better than humans?. Front. Cell. Neurosci..

[44] Klppel S (2009). Functional compensation of motor function in pre-symptomatic Huntingtons disease. Brain.

[45] Cameron, M. et al. Calcium imaging of AM dyes following prolonged incubation in acute neuronal tissue. *PLoS ONE* **11**, e0155468 (2016). 10.1371/journal.pone.0155468 PMC4868260 27183102

[46] Breen P. P. & Buskila, Y. Braincubator: An incubation system to extend brain slice lifespan for use in neurophysiology. *2014 36th Annual International Conference of the IEEE Engineering in Medicine and Biology Society*, Chicago, IL, pp. 4864-4867. (2014) 10.1109/EMBC.2014.6944713 25571081

[47] Buskila Y (2014). Extending the viability of acute brain slices. Sci. Rep..

[48] Cameron MA (2017). Prolonged incubation of acute neuronal tissue for electrophysiology and calcium-imaging. J. Vis. Exp..

[49] Buskila Y, Amitai Y (2010). Astrocytic iNOS-dependent enhancement of synaptic release in mouse neocortex. J. Neurophysiol..

[50] Spandidos A, Wang X, Wang H, Seed B (2009). PrimerBank: a resource of human and mouse PCR primer pairs for gene expression detection and quantification. Nucleic Acids Res..

