# Supplementary material for: Dynamic interplay between H-current and M-current controls motoneuron hyperexcitability in amyotrophic lateral sclerosis
Source: Cell Death Dis. 2019 Apr 5;10(4):310. doi: 10.1038/s41419-019-1538-9 (PMC6450866; doi:10.1038/s41419-019-1538-9)
Supplement: Supplementary file 2 — supplementary figure legends [file 41419_2019_1538_MOESM2_ESM.docx]

**Dynamic interplay between H-current and M-current controls motoneuron hyperexcitability in amyotrophic lateral sclerosis**

Yossi Buskilaa,b*, Orsolya Kékesia,b,c,d, Alba Bellot-Saeza,b, Winston Seaha,b,c,d, Tracey Bergc,d, Michael Trpceskic,d, Justin J. Yerburyc,d and Lezanne Ooic,d*

***Supplementary figure 1****.* Differentiation of motoneurons based on the Sag amplitude. A) sample traces of two types of layer 5b pyramidal neurons expressing ‘high Sag’ (left) and ‘low Sag’ (right) profiles. B) Box plots depicting the distribution of Sag amplitudes normalized to the overall deflection of the membrane potential. The box upper and lower limits are the 25th and 75th quartiles, respectively. The whiskers depicting the lowest and highest data points, while the **+** sign represents the mean and the horizontal line through the box is the median.
